# Supplementary material for: Survival rates of adult and juvenile gyrfalcons in Iceland: estimates and drivers
Source: PeerJ. 2021 Nov 23;9:e12404. doi: 10.7717/peerj.12404 (PMC8621780; doi:10.7717/peerj.12404)
Supplement: Supplemental Information 1 [file peerj-09-12404-s001.pdf]

Supporting Information for *Survival rates of adult and juvenile gyrfalcons in Iceland: estimates and drivers* by Frédéric Barraquand and Ólafur K. Nielsen

Supplementary tables

Statistic	Prey abundance	Temperature	Log-precipitation
Mean	6.080	-0.897	3.671
SD	2.487	0.958	0.257

Table S1. Mean and SD of the three covariates.

Parameter	Mean	SE	SD	2.5%	25%	50%	75%	97.5%	n_{eff}	$\hat{R}$
s_2	0.834	0.000	0.018	0.797	0.822	0.834	0.846	0.868	3095	1.000
η	0.009	0.000	0.009	0.000	0.003	0.006	0.012	0.031	3702	0.999
r	0.139	0.000	0.009	0.123	0.134	0.139	0.145	0.157	3314	1.000
p	0.030	0.000	0.005	0.021	0.026	0.029	0.033	0.040	2382	0.999
β_1	0.281	0.003	0.134	0.022	0.190	0.283	0.370	0.542	1859	1.000
β_2	0.230	0.003	0.121	-0.007	0.151	0.230	0.310	0.474	2093	1.000
β_3	0.272	0.003	0.127	0.027	0.185	0.269	0.362	0.517	2082	1.000
μ_{s_1}	-0.518	0.002	0.115	-0.741	-0.594	-0.517	-0.442	-0.293	2296	1.000

Table S2. Estimates of the Mark-Recovery-Recapture model with 3 covariates on the juvenile survival rate s_1. Parameters: s_2 adult survival rate, η carcass survival rate, r recovery probability, p recapture/resighting probability, β_1 effect of ptarmigan abundance, β_2 effect of temperature, β_3 effect of log-precipitation, μ_{s_1} intercept in the logistic regression.

Priors	A	B	C	S
Uniform	0.00	-0.81	-0.16	-66.48
Beta	0.00	-0.95	-0.09	-66.56

Table S3. Differences in expected predictive log-density according to PSIS-LOO.

Supplementary figures

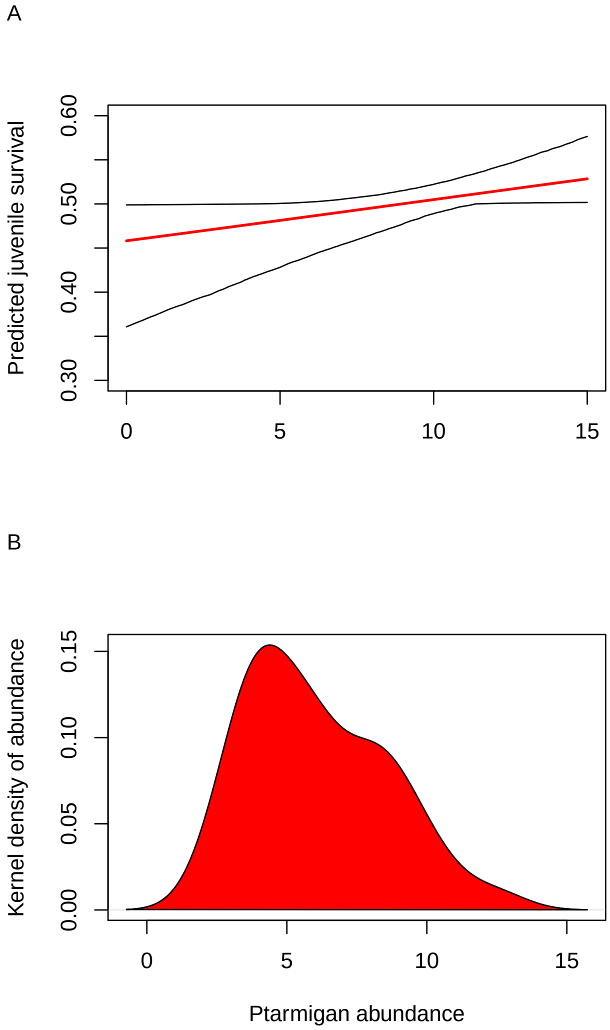

Figure S1. Ptarmigan density effect on juvenile survival with an informative prior (positivity constraint on the slope of the logistic regression). An a priori mean slope of 1/100 with exponential distribution is assumed; hence we assume ptarmigan effect to be weak before model fitting. The effect of this parameterization is to move up the juvenile survival values, which may be artefactual.

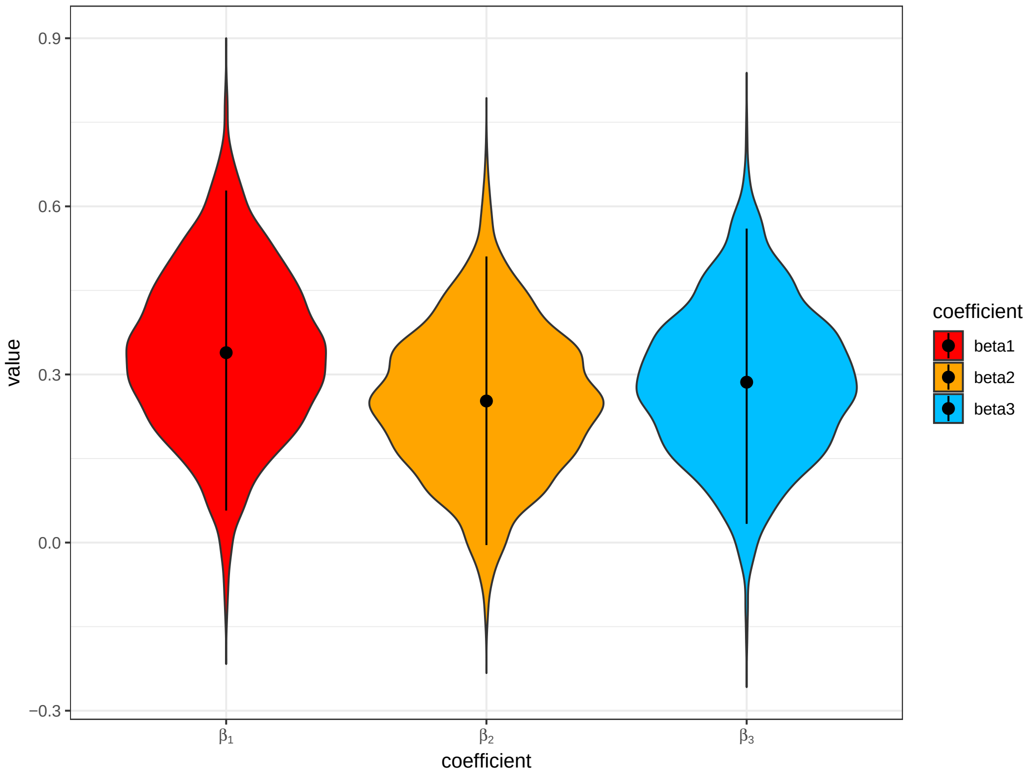

Figure S2. Violin plots for model C coefficient estimates.
